# Clinical Benefits of Olaparib in Mexican Ovarian Cancer Patients With Founder Mutation *BRCA1*-Del ex9-12

**DOI:** 10.3389/fgene.2022.863956

**Published:** 2022-06-06

**Authors:** Dolores Gallardo-Rincón, Edgar Montes-Servín, Gabriela Alamilla-García, Elizabeth Montes-Servín, Antonio Bahena-González, Lucely Cetina-Pérez, Flavia Morales Vásquez, Claudia Cano-Blanco, Jaime Coronel-Martínez, Ernesto González-Ibarra, Raquel Espinosa-Romero, Rosa María Alvarez-Gómez, Abraham Pedroza-Torres, Denisse Castro-Eguiluz

**Affiliations:** ^1^ Ovarian and Endometrial Cancer Program (COE), Instituto Nacional de Cancerología (INCan), Mexico City, Mexico; ^2^ Department of Medical Oncology, Instituto Nacional de Cancerología (INCan), Mexico City, Mexico; ^3^ Department of Clinical Research and Medical Oncology, Instituto Nacional de Cancerología (INCan), Mexico City, Mexico; ^4^ Cervical Cancer Program (Micaela), Instituto Nacional de Cancerología (INCan), Mexico City, Mexico; ^5^ Hereditary Cancer Clinic, Instituto Nacional de Cancerología (INCan), Mexico City, Mexico; ^6^ Catedrático CONACYT, Instituto Nacional de Cancerología, Mexico City, Mexico

**Keywords:** epithelial ovarian cancer, Mexican founder mutation, large rearrangements, progression-free survival, *BRCA* mutation

## Abstract

**Background:** Ovarian cancer (OC) is gynecologic cancer with the highest mortality rate. It is estimated that 13–17% of ovarian cancers are due to heritable mutations in *BRCA1* and *BRCA2*. The *BRCA1* (*BRCA1*-Del ex9-12) Mexican founder mutation is responsible for 28–35% of the cases with ovarian cancer. The aim was to describe the PFS of OC patients treated with olaparib, emphasizing patients carrying the Mexican founder mutation (*BRCA1*-Del ex9-12).

**Methods:** In this observational study, of 107 patients with *BRCA*m, 35 patients were treated with olaparib from November 2016 to May 2021 at the Ovarian Cancer Program (COE) of Mexico; patient information was extracted from electronic medical records.

**Results:** Of 311 patients, 107 (34.4%) were with *BRCA*m; 71.9% (77/107) were with *BRCA1*, of which 27.3% (21/77) were with *BRCA1*-Del ex9-12, and 28.1% (30/107) were with *BRCA2* mutations. Only 35 patients received olaparib treatment, and the median follow-up was 12.87 months. The PFS of *BRCA1*-Del ex9-12 was NR (non-reach); however, 73% of the patients received the treatment at 36 vs. 11.59 months (95% CI; 10.43–12.75) in patients with other *BRCA*m (*p* = 0.008). Almost 50% of patients required dose reduction due to toxicity; the most frequent adverse events were hematological in 76.5% and gastrointestinal in 4%.

**Conclusion:** Mexican OC *BRCA1*-Del ex9-12 patients treated with olaparib had a significant increase in PFS regardless of the line of treatment compared to other mutations in *BRCA*.

## Introduction

Among gynecologic cancers, ovarian cancer (OC) has the highest mortality rate. Epithelial ovarian cancer is the most lethal gynecologic malignancy, as it is commonly diagnosed at an advanced stage and only 10% of all OC is non-epithelial (include mainly germ cell tumors, sex cord–stromal tumors, and some rare tumors) (Boussios et al., 2017). According to GLOBOCAN estimates, in 2020, there were 313,959 new cases and 207,252 deaths worldwide. In Mexico, the estimated number of new cases and deaths for the same year were 4,963 and 3,038, respectively (The Global Cancer Observatory, 2020).

The recent addition of poly (ADP-ribose) polymerase (PARP) inhibitor (PARPi) as a treatment option has caused a paradigm shift in the management of OC patients. PARP prevents the repair of single-stranded DNA breaks and, coupled with a deficiency in repair by homologous recombination, causes synthetic lethality and cell death (Weaver and Yang, 2013). Olaparib, niraparib, and rucaparib are novel oral PARPi agents that have become a standard of care in different clinical settings, such as maintenance therapy after platinum-sensitive recurrence with either partial or complete response or after frontline therapy. Although clinical trials have demonstrated the efficacy of PARPi in the absence of homologous recombination deficiency, patients with *BRCA* gene mutations achieve better outcomes (Coleman et al., 2017; Del Campo et al., 2019; González-Martín et al., 2019). However, apart from mutations in the *BRCA1/2* genes, there are other genomic alterations involving genes in homologous recombination pathways like the Fanconi anemia genes (*BRIP1* and *PALB2*), the core RAD genes (*RAD51C* and *RAD51D*), and genes involved directly (*CHEK2*, *BARD1, NBN*, and *ATM*) or indirectly (*CDK12*). The genome-wide association studies identified single-nucleotide polymorphisms associated with susceptibility for epithelial OC, for example, 27 loci are associated with invasive epithelial OC identified so far account for 6.4% of the polygenic risk for epithelial OC (Boussios et al., 2020).

Mutations in *BRCA1/2* occur in 1 out of 300–500 women, increasing their risk of developing various types of cancer, predominantly breast and ovarian cancer (Zhang et al., 2011; Toss et al., 2015). It is estimated that 13–17% of OC are due to heritable mutations in *BRCA1* and *BRCA2* (Hennessy et al., 2010; Cancer Genome Atlas Research Network, 2011). In addition, 3–7% of OC patients harbor a somatic mutation of the *BRCA* genes (Cunningham et al., 2014; Pennington et al., 2014). There are previous reports of *BRCA* mutation frequency in Mexican OC patients. The first study carried out by Villarreal-Garza et al. (2015a) tested *BRCA* mutations (using HISPANEL) in 188 non-related patients (92 with OC and 96 with breast cancer (BC)). This study reported that *BRCA* mutations were detected in 28% of OC patients and most of the mutations were in *BRCA1* (88%). Gallardo-Rincón et al. (2020) studied 179 OC patients for germline *BRCA* mutations through next-generation sequencing and multiplex ligation-dependent probe amplification. In this study, 33% of patients had a germline mutation and 66% of these were found in *BRCA1*. In addition, the most frequent mutation for Mexican *BRCA* mutation carriers was the deletion of exons 9 to 12 in *BRCA1* (*BRCA1*-Del ex9-12) representing the 28% (11/39) of *BRCA1*-mutated patients. Other studies support these data in BC and OC patients combined (Vaca-Paniagua et al., 2012; Quezada Urban et al., 2018; Oliver et al., 2019).

The *BRCA1* Mexican founder mutation (*BRCA1*-Del ex9-12 or NM_007294.3: c.548-?_4,185+?del) is related to a clear founder effect (Weitzel et al., 2005; Weitzel et al., 2007; Weitzel et al., 2013). The previously mentioned epidemiological studies reported that the founder mutation accounts for 28–35% of *BRCA* gene mutations in Mexican OC (Cunningham et al., 2014; Villarreal-Garza et al., 2015a). The Mexican founder mutation is a large rearrangement (exon deletion). Previous reports suggest the possibility that large rearrangements represent a type of *BRCA* gene mutation with greater penetrance for cancer risk, as it correlates with earlier onset age or more aggressive tumors in BC and OC patients (James et al., 2015; Kwong et al., 2015). This molecular feature could have a meaningful clinical impact on screening, prognosis, and treatment in the case of PARPi. The aim of this study was to describe the survival rate of OC patients treated with olaparib, emphasizing patients carrying the Mexican founder mutation (*BRCA1*-Del ex9-12).

## Materials and Methods

### Study Design

In this single-center observational study, data analysis was carried out from retrospectively collected samples with prospectively followed up. A total of 311 OC patients in clinical stages (CS) from IA to IVB were enrolled from October 2015 to May 2021 at the Instituto Nacional de Cancerología (INCan) of Mexico. All patients provided written informed consent before entering the study. Of 311 OC patients, 35 were treated with olaparib at the Ovarian and Endometrial Cancer Program (COE) at INCan, from November 2016 to May 2021.

### Patients

Patient inclusion criteria were: 1. histopathology confirmed diagnosis of epithelial ovarian cancer platinum-sensitive, at any clinical stage. 2. *BRCA1/2* germinal mutation. 3. Partial or complete objective response (either according to response evaluation criteria in solid tumors (RECIST) version 1.1 or patients with stable disease with a decreased level of CA-125 4 of olaparib maintenance treatment starting 4–8 weeks after the last chemotherapy cycle. In November 2020, the first-line olaparib maintenance treatment in OC patients was started. Olaparib dose was 800 mg/day, as maintenance therapy until progression; dose adjustment administration was allowed in grade 2 or more adverse events.

### Study Endpoints and Assessments

The primary objective was to evaluate the benefit of olaparib in Mexican OC patients by describing their PFS according to *BRCA1* founder mutation. PFS was defined as the time from the beginning of treatment with olaparib to disease progression, death, or the last contact at a cutoff date of 31 May 2021. Baseline clinicopathological characteristics such as age, histology, stage assigned at diagnosis, and toxicity were extracted from electronic medical records.

### Statistical Analyses

Continuous variables were tabulated as medians with ranges or as means with standard deviations (SDs), depending on the data distribution. The distribution was assessed using the Shapiro–Wilk test with a *p*-value greater than 0.05 considered as normally distributed. Two-group comparisons were tested using Student’s t-test or Mann–Whitney U test depending on the data distribution. Nominal data were analyzed using the chi-squared (X^2^) test. Median PFS curves were estimated using the Kaplan–Meier method, while comparisons among groups were analyzed with log-rank or Breslow tests. Statistical significance was determined as *p* ≤ 0.05 with a two-sided test. All data were analyzed using the SPSS software package version 26 (SPSS, Inc., Chicago, Ill, United States) and GraphPad Prism version 9.0 (GraphPad San Diego, CA, United States).

## Results

### Presence of Germinal *BRCA* Mutations

**FIGURE 1:**
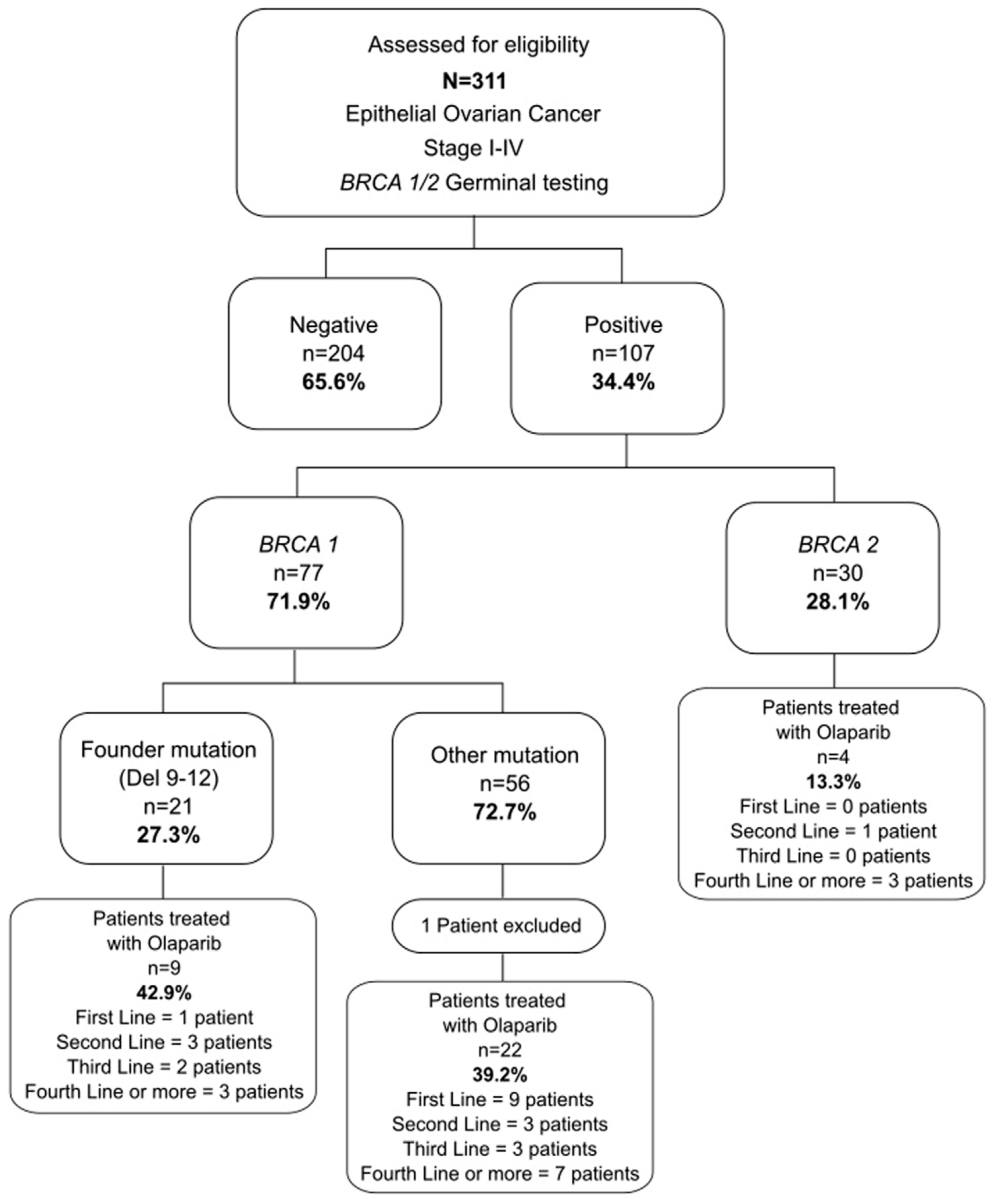
Patient enrollment flowchart. Flowchart summarizes patient enrollment and sub-analysis groups.

Of 311 OC patients, 107 (34.4%) had a germinal *BRCA*m, of which 71.9% (77/107) were *BRCA1* and 28.1% (30/107) were *BRCA2* mutations. Among these patients, the most common pathogenic variant detected in 21 (27.3%) was *BRCA1*-Del ex9-12 (Mexican founder mutation). The patient enrollment, testing flowchart, and line of treatment subgroups are summarized in Figure 1.

### Patient Characteristics

**FIGURE 2:**
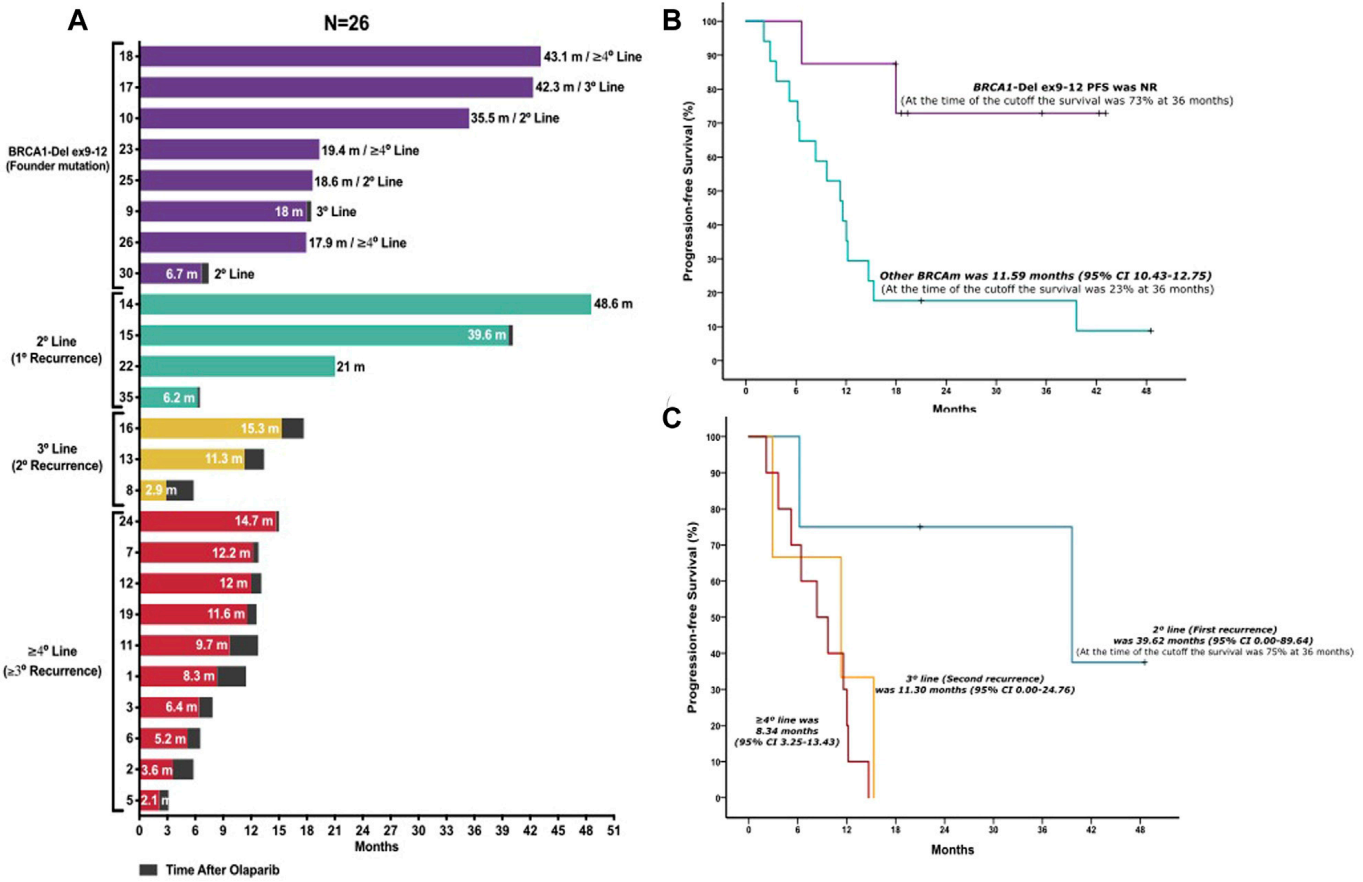
Patient PFS analysis. **(A)** Histogram per patient shows the follow-up of each patient undergoing olaparib treatment and time after progression. Patients are grouped according to the presence of the Mexican founder mutation and other mutations organized by the lines of treatment received before olaparib maintenance therapy. Patients with *BRCA1* founder mutation (*BRCA1*-Del ex9-12) (purple bars); patients with other *BRCA* mutations: second line (turquoise bars); third line (yellow bars); and patients in fourth line or more (red bars). **(B)** Progression-free survival comparison between *BRCA1*-Del ex9-12 and other *BRCA* mutations of patients undergoing olaparib maintenance therapy. Kaplan–Meier curve of PFS. Patients with *BRCA1* founder mutation (*BRCA1*-Del ex9-12) (purple line); patients with other *BRCA* mutations (turquoise line). **(C)** Progression-free survival comparison between lines of treatment of the other *BRCA* mutations of patients undergoing olaparib maintenance therapy. Kaplan–Meier curve of PFS. Patients in second line (turquoise line), third line (yellow line), and patients in fourth line or more (red line).

A total of 36 patients received olaparib, and only 35 patients were analyzed in this study; one patient was excluded because she received gemcitabine instead of platinum-based chemotherapy before olaparib treatment (platinum hypersensitivity). The median age was 51 years (range, 40–69); 33 patients were with HGSP histology (94.2%), and 19 patients (54.3%) had a clinical-stage IIIC disease. Most of the patients did not present comorbidities like diabetes mellitus and systemic arterial hypertension (88.6 and 82.9%, respectively). Patients confirmed with the first and second grade of cancer family history (CFH) were 31 (88.6%), 12 patients (34.3%) for OC and 23 patients (65.7%) for BC, and 15 patients (42.9%) were positive for other *BRCA*-associated cancer types (prostate, pancreatic, and gastrointestinal). Almost 23% (8 patients) had double primary malignancy (breast–ovarian) (Table 1). The median of follow-up of the 35 patients was 12.87 months. By the time of data analysis, 21 patients (60%) had disease progression to olaparib maintenance therapy, and 14 patients (40%) were still receiving olaparib treatment (Figure 2A). There were no statistical differences in the clinical characteristics according to the *BRCA*m, which are visualized in Table 1. Genetic variants of *BRCA1/2* were classified according to the prevalence in the cohort of patients, the ovarian cancer cluster regions (OCCRs), and breast cancer cluster regions (BCCRs) in both genes (*BRCA1/2*) Table 2.

**TABLE 1:** Baseline characteristics.

Variable	Total % (n = 35)	*BRCA1*	Other *BRCA* mutation	P^a^
		(Founder mutation)		
		% (n = 9)	% (n = 26)	
**Age at diagnosis (years)**				
Mean ± S. D	51.31 ± 7.38	50 ± 7.08	51.77 ± 7.57	0.544
Median (range)	51 (40–69)	52 (40–63)	51 (40–69)	0.677
**Histology**				
HGS	94.2 (33/35)	100 (9/9)	92.4 (24/26)	0.693
Adenocarcinoma	2.9 (1/35)	0 (0 (9)	3.8 (1/26)	
Others	2.9 (1/35)	0 (0 (9)	3.8 (1/26)	
**Stage**				
I–II	11.4 (4/35)	0 (0/9)	15.4 (4/26)	0.084
IIIA–B	8.6 (3/35)	22.22 (2/9)	3.8 (1/26)	
IIIC	54.3 (19/35)	33.33 (3/9)	61.5 (16/26)	
IV	25.7 (9/35)	44.44 (4/9)	19.2 (5/26)	
**DM**				
Negative	88.6 (31/35)	100 (9/9)	84.6 (22/26)	0.211
Positive	11.4 (4/35)	0 (0 (9)	15.4 (4/26)	
**SAH**				
Negative	82.9 (29/35)	88.9 (8/9)	80.8 (21/26)	0.577
Positive	17.1 (6/35)	11.1 (1/9)	19.2 (5/26)	
**CFH**				
Negative	11.4 (4/35)	0 (0/9)	15.4 (4/26)	0.211
Positive	88.6 (31/35)	100 (9/9)	84.6 (22/26)	
**Ovarian CFH**				
Negative	65.7 (23/35)	77.8 (7/9)	61.5 (16/26)	0.376
Positive	34.3 (12/35)	22.2 (2/9)	38.5 (10/26)	
**Breast CFH**				
Negative	34.3 (12/35)	11.1 (1/9)	42.3 (11/26)	0.089
Positive	65.7 (23/35)	88.9 (8/9)	57.7 (15/26)	
**Other CFH**				
Negative	57.1 (20/35)	44.4 (4/9)	61.5 (16/26)	0.372
Positive	42.9 (15/35)	55.6 (5/9)	38.5 (10/26)	
Pancreatic	26.7 (4/15)	40 (2/5)	20 (2/10)	0.638
Prostate	33.3 (5/15)	20 (1/5)	40 (4/10)	
Gastrointestinal	40 (6/15)	40 (2/5)	40 (4/10)	
**Double primary malignancy**				
Negative	77.1 (27/35)	55.6 (5/9)	84.6 (22/26)	0.074
Positive (breast–ovarian)	22.9 (8/35)	44.44 (4/9)	15.4 (4/26)	
**CA-125 at starting treatment line (U/mL)**				
Mean ± S. D	1009.91 ± 2177.25	2099.08 ± 3968.29	632.88 ± 912.66	0.303
Median (range)	205 (17.40–9244.70)	108.60 (17.40–9244.70)	245.45 (28.00–4,019.40)	0.521
**CA-125 at start olaparib (U/mL)**				
Mean ± S. D	24.40 ± 32.83	13.80 ± 6.08	21.20 ± 42.63	0.250
Median (range)	14.30 (5.60–194.40)	13.80 (9.50–18.10)	32.47 (6.11–194.40)	0.664
**CA-125 at progression to olaparib (U/mL)**				
Decrease	133.28 ± 248.76	12.25 ± 5.86	146.02 ± 258.76	**0.037**
Increase	52.10 (8.10–1092.30)	12.25 (8.10–16.40)	56.10 (10.90–1092.30)	0.072
**Δ CA-125 (treatment-olaparib)**				
Decrease	100 (35/35)	100 (9/9)	100 (26/26)	--
Increase	0 (0/35)	0 (0/9)	0 (0/26)	
**Δ CA-125 (olaparib-progression)**				
Decrease	19 (4/21)	100 (2/2)	10.5 (2/19)	**0.002**
Increase	81 (17/21)	0 (0/2)	89.5 817/19)	

a Chi-squared (X^2^) test.

Abbreviations: CBP: carboplatin; TXL: taxol; CDDP: cisplatin; GMZ: gemcitabine; BVZ: bevacizumab; HGS: high-grade serous; DM: diabetes mellitus; SAH: systemic arterial hypertension; CFH: cancer family history.

Note: Bold numbers are statistically significant values

**TABLE 2:** *BRCA* mutation

Patient ID	Variant/population(s) reported	Cluster region	Gene
36	**Del ex9-12/(AP: Mexican founder mutation)**	**OCCR**	***BRCA1***
30			
10			
25			
17			
9			
26			
23			
18			
32	c.1960A > T (p. Lys654Ter)		
8			
1			
14	**c.1674del (p. Gly559fs)**		
	**(AP: Colombian founder mutation)**		
34	c.211A > G (p. Arg71Gly)		
21	c.2296_2297del (p.Glu765_Ser766insTer)		
2	c.2611+1G > T		
7	c.3598 C > T (p. Gin1200*)		
28	c.3759_3760del (p. Lys1254fs)		
20	c.4327C > T (p. Arg1443Ter)		
16	c.5123C > A (p. Ala1708Glu)		
33	c.5165C > T (p. Ser1722Phe)		
31	c.5278-1G > C		
27	c.4868 C > G (p. ALA1623GLY)	**BCCR**	
13			
5			
22	**c.68_69del (p. Glu23fs)**		
24	**(AP: Ashkenazi-Jewish)**		
29	c.1504_1508del (p. Leu502fs)		
35	c.68_69dupAG (p. Cys24Serfs)		
11	c.815_824dup (p. Thr276fs)		
6	Del ex18-19		
3	c.5616_5620delAGTAA (p. Lys1872Asnfs)	**OCCR**	***BRCA2***
15	c.5631delC (p. Asn1877Lysfs)		
19	c.6352_6353delGT (p. Val2118Lysfs)	**BCCR**	
12	c.8168A > G (p. Asp2723Gly)		

Abbreviations: OCCRs: ovarian cancer cluster regions; BCCRs: breast cancer cluster regions; AP: associated population.

Note: Bold numbers are statistically significant values

### Olaparib Maintenance Therapy Patient Characteristics

In total, 35 patients received olaparib maintenance therapy after platinum-based chemotherapy; 91.4% had a complete or partial response (n = 32), and 3 patients had stable disease (8.6%) before starting olaparib maintenance therapy. The number of patients treated with olaparib after the first line was 10 (27.8%) with a mean follow-up of 10.55 months. The platinum-sensitive, relapsed patients treated with olaparib at the second line (first recurrence) were 7 (20%), 5 patients (14.3%) at the third line (second recurrence), and 13 patients at fourth or more line of treatment (37.1%), all with a mean follow-up of 17.29 months.

Analyzing the patients with recurrent disease (first-line treated patients excluded), a platinum-free interval status was evaluated in 26 patients, 40% (14 patients) had a response of 6–12 months, and 31.4% (11 patients) had a response of 12 months or higher. Most of the mutations (68.6%) are located in the areas known as the OC cluster regions (OCCRs), and 31.4% are located in the BC cluster regions (BCCRs) in both genes (*BRCA1/2*). There were no statistical differences in the clinical characteristics of patients that received olaparib maintenance therapy according to the *BRCA*m which are visualized in S1.

### Progression-Free Survival Analysis

The median follow-up of the 35 patients was 12.8 months (95% CI 8.82–16.92). The only baseline characteristic associated with olaparib PFS was breast CFH; these patients had a better survival (11.59 vs. 17.97 months *p* = 0.036). There were no statistical differences in the baseline characteristics associated with *BRCA*m as shown in S2. The median PFS of positive founder mutation *BRCA1*-Del ex9-12 was NR (at the time of cutoff, the survival was 73% at 36 months) vs. 11.59 months (95% CI 10.43–12.75) in those with other *BRCA*m detected (*p* = 0.008) (Figure 2B and Supplementary Figure S3). The PFS from the patients with positive founder mutation *BRCA1*-Del ex9-12 shows a significant increase regardless of the line of treatment in which they received the treatment compared to other mutations in *BRCA*. The median PFS of other *BRCA*m detected treated with olaparib after the first line was 12.87 months; also, 39.62 months for the patients treated at the second line (first recurrence), 11.30 months for patients at the third line (second recurrence), and 8.34 months for patients at the fourth or more lines of treatment were reported (Figure 2C and Supplementary Figure S3). The group of other *BRCA* mutations showed that patients with a complete or partial response before olaparib maintenance therapy had a better PFS than patients with stable disease (*p* = 0.008). Also, multi-treated patients (≥4°L) had the worst PFS among the other lines of treatment (*p* = 0.029) (S3).

### Toxicity

Toxicity adverse events were obtained from the 35 analyzed patients. Of all patients, 48.6% (17/35) required dose reduction due to some adverse event of any grade (most of these patients were in the third or more lines of treatment). The most frequent adverse events in the patients with dose reduction were hematological in 76.5% (13 patients) and gastrointestinal in 23.5% (4 patients) (S1).

The use of olaparib was associated with neutropenia in 1 case with grade 3 and anemia in 15 patients (42.8%). Grade 2 anemia in 33.4% (5/15 patients), grade 3 in 46.6% (7/15 patients), and grade 4 in 20% (3/15 patients) were developed. On the other hand, 75% (3/4) of the patients had nausea grade 2 (2 patients), grade 3 in 1 patient, and 1 patient with dysgeusia grade 2. Other adverse event recorded in this cohort was pneumonitis in only 1 case associated with previous breast radiotherapy treatment. Expected adverse events related to the use of olaparib, such as myelodysplastic syndrome (MDS), occurred in 1 patient (toxicity events by subgroups are summarized in S4).

## Discussion

The *BRCA1*-Del ex9-12 mutation is related to a founder effect in the Mexican population. Epidemiological studies reported that this founder mutation represents the 28–39% of *BRCA1* gene mutations in Mexican OC patients. In addition, another frequent mutation was present in the OCCR, *BRCA1* c.1970A > T (p.Lys654Ter) at 8.6%. This mutation predicts loss of normal protein function through either protein truncation or nonsense-mediated mRNA decay (Judkins et al., 2005); Weitzel et al. (2005) reported that this mutation is associated with a high risk of developing cancer and is considered a frequent mutation in the Mexican population. The most common mutation in the BCCR of the *BRCA1* gene is c.4868C > G (p.Ala1623Gly) at 8.6%, which is associated with a partial deletion in the exon 15, which is a rare mechanism of splicing alteration (Byers et al., 2016). This specific mutation is associated with a risk of more aggressive breast cancer in men, but its effect in ovarian cancer patients is unknown (Alsop et al., 2012).

Other founder effects have been reported in Latin American countries, such as Brazil (*BRCA1* 5382insC and *BRCA2* c.156_157insAlu) and Colombia (*BRCA1* 3450del4, *BRCA1* A1708E, and *BRCA2* 3034del4) (Ossa and Torres, 2016). Of these, *BRCA1* 3450del4 mutation has also been reported in Brazil and Chile, whereas mutation *BRCA2* 3034del4 has been reported in Argentina and Peru. These data imply that Hispanic (Latin American) populations share common genetic ancestry components from Europe, Africa, and Native Americans which are also genetically heterogeneous (Bryc et al., 2010). To our knowledge, this is the first report of the association of these specific mutations with survival and other outcomes in OC patients.

In our populations, most of the mutations detected in *BRCA* genes were point mutations. The *BRCA1*-Del ex9-12 mutation and *BRCA1*-Del ex18-19 represent the only cases of large rearrangement (exon deletion). Large gene rearrangements (LGRs) represent less than 10% of *BRCA1* pathogenic variants (Sluiter and van Rensburg, 2011). Latin American patients report a prevalence of nearly 21%, similar to Dutch (27%) and Italian (20%) populations (Judkins et al., 2012). We identified that *BRCA1*-Del ex18-19 was detected in a single patient. LGRs in the *BRCA* gene are associated with greater penetrance for cancer risk and correlate with an earlier onset age of cancer or more aggressive tumors (James et al., 2015). Due to the large-scale sequencing efforts, there is currently a better understanding of the genomic landscape of several malignancies, for example, the incidence of germline *BRCA* mutations in newly diagnosed prostate cancer patients is 1.2–2%, and the *BRCA1/2* carriers can have around 4- and 8-fold risk of developing prostate cancer, respectively. So, the importance of detection and the identification of defects in DNA repair genes have led to clinical studies that provide a strong rationale for developing PARPi and DNA-damaging agents in this molecularly defined subset of patients (Ghose et al., 2021).

It has been demonstrated that triple-negative breast cancer patients with the Mexican founder mutation have the worst outcome (Villarreal-Garza et al., 2015b). In agreement with this report; of eight patients that present a double primary malignant neoplasm, four patients were founder-mutated with triple-negative breast cancer and two patients had progressive disease. Probably, the main reason regarding the difference in survival between patients with small-scale *BRCA* mutations and LGRs relies on the resistance mechanisms to PARPi, such as olaparib. Among the most important is mutational reversion, restoring homologous recombination repair of DNA double-strand breaks (Banda et al., 2018).

In this way, *BRCA1* 9–12 exon deletion represents the loss of more than 60% of the gene-coding region, so it would be complex to opt for a mutational reversion resistance mechanism to restore the wild-type allele and thus correlate with the better response and survival. Similar observations have been reported in patients with *BRCA* LGRs and their response to platinum and PARPi (Randall et al., 2020; Wang et al., 2022).

The median PFS reported in the SOLO2 trial in first platinum-sensitive, relapsed *BRCA*-mutated OC patients treated with olaparib was higher (19.1 months [95% CI 16.3–25.7]) than that in the placebo (5.5 months [5.2–5.8]; hazard ratio [HR] 0.30 [95% CI 0.22–0.41], *p* < 0.0001) with 22.1 months of follow-up (Pujade-Lauraine et al., 2017); in our study, the median PFS for the first recurrent platinum-sensitive OC patients like the SOLO2 trial patient characteristics (n = 25) was 39.68 months with a median follow-up of 17.2 months.

The median PFS reported in the SOLO1 trial in first-line maintenance therapy OC patients with *BRCA1/2* mutation was 60% at 3 years of follow-up (Moore et al., 2018); in our study, the median PFS for positive Mexican founder mutation in *BRCA1* was NR, but 73% at 3 years vs. 11.59 months (95% CI 10.43–12.75) in those with other *BRCA*m were detected. (*p* = 0.004). Our results are consistent and confirmed the preliminary results that were reported by Gallardo-Rincón et al. (2019).

Regarding the use of olaparib in the first line of treatment, our data are immature, and we are still recruiting patients with the founder mutation to compare their survival with other *BRCA* mutations. At the time of the cut-off, 10 patients were receiving maintenance treatment after the first line, and the median PFS was 12.87 months with a short follow-up of 10.5 months. *BRCA1*-Del ex9-12-mutated patients that required dose reduction reported fewer adverse events associated with olaparib treatment than other *BRCA*m patients (33 vs. 52%, respectively). Multi-treated patients (≥4°L) reported more and higher toxicity effects.

Despite the limited number of patients that received olaparib treatment (N = 35), the obtained results are precise on its clinical benefit for patients with PARPi, especially for patients with *BRCA1*-Del ex9-12 Mexican founder mutation. We consider that a new prospective study would be feasible and essential because it may provide more evidence on the efficacy of PARPi in this patient population. Therefore, we recommend the detection of the founder mutation in patients susceptible to treatment with PARPi since the patients in our study benefited from olaparib.

In Mexico, mutation screening in OC patients with and without cancer family history is limited (Martínez-Treviño et al., 2018). Very few research and medical oncology care centers provide this multidisciplinary care service, which allows the identification of patients that may benefit from new therapies for treatment with PARPi (Fragoso-Ontiveros et al., 2019). We highlighted the need to include genetic risk assessment and molecular testing in medical oncology centers that also allows genetic counseling to detect this Mexican founder mutation at diagnosis due to its prevalence in the OC patient population. Therefore, based on our results, we propose that the mutation status (*BRCA1-*Del ex9-12) should be an additional stratification factor in the standard treatment of patients.

The Mexican OC patients with the founder mutation (*BRCA1*-Del ex9-12), treated with PARPi maintenance therapy (olaparib), show a significant increase in PFS regardless of the line of treatment compared to other mutations in *BRCA*.

## Data Availability

The original contributions presented in the study are included in the article/Supplementary Material; further inquiries can be directed to the corresponding author.

## References

[B1] Alsop K. Fereday S. Meldrum C. deFazio A. Emmanuel C. George J. (2012). BRCA Mutation Frequency and Patterns of Treatment Response inBRCA Mutation-Positive Women with Ovarian Cancer: A Report from the Australian Ovarian Cancer Study Group. Jco 30 (21), 2654–2663. 10.1200/jco.2011.39.8545 PMC3413277 22711857

[B2] Banda K. Swisher E. M. Wu D. Pritchard C. C. Gadi V. K. (2018). Somatic Reversion of Germline BRCA2 Mutation Confers Resistance to Poly(ADP-Ribose) Polymerase Inhibitor Therapy. JCO Precis. Oncol. 2, 1–6. 10.1200/po.17.00044 35135104

[B3] Boussios S. Moschetta M. Zarkavelis G. Papadaki A. Kefas A. Tatsi K. (2017). Ovarian Sex-Cord Stromal Tumours and Small Cell Tumours: Pathological, Genetic and Management Aspects. Crit. Rev. Oncology/Hematology 120, 43–51. 10.1016/j.critrevonc.2017.10.007 29198337

[B4] Boussios S. Mikropoulos C. Samartzis E. Karihtala P. Moschetta M. Sheriff M. (2020). Wise Management of Ovarian Cancer: On the Cutting Edge. Jpm 10 (2), 41. 10.3390/jpm10020041 PMC7354604 32455595

[B5] Bryc K. Velez C. Karafet T. Moreno-Estrada A. Reynolds A. Auton A. (2010). Colloquium Paper: Genome-wide Patterns of Population Structure and Admixture Among Hispanic/Latino Populations. Proc. Natl. Acad. Sci. U. S. A. 107 (Suppl. 2), 8954–8961. 10.1073/pnas.0914618107 20445096 PMC3024022

[B6] Byers H. Wallis Y. van Veen E. M. Lalloo F. Reay K. Smith P. (2016). Sensitivity of BRCA1/2 Testing in High-Risk Breast/ovarian/male Breast Cancer Families: Little Contribution of Comprehensive RNA/NGS Panel Testing. Eur. J. Hum. Genet. 24 (11), 1591–1597. 10.1038/ejhg.2016.57 27273131 PMC5110056

[B7] Cancer Genome Atlas Research Network (2011). Integrated Genomic Analyses of Ovarian Carcinoma. Nature 474 (7353), 609–615. 10.1038/nature10166 21720365 PMC3163504

[B8] Coleman R. L. Oza A. M. Lorusso D. Aghajanian C. Oaknin A. Dean A. (2017). Rucaparib Maintenance Treatment for Recurrent Ovarian Carcinoma after Response to Platinum Therapy (ARIEL3): a Randomised, Double-Blind, Placebo-Controlled, Phase 3 Trial. Lancet 390 (10106), 1949–1961. 10.1016/S0140-6736(17)32440-6 28916367 PMC5901715

[B9] Cunningham J. M. Cicek M. S. Larson N. B. Davila J. Wang C. Larson M. C. (2014). Clinical Characteristics of Ovarian Cancer Classified by BRCA1, BRCA2 and RAD51C Status. Sci. Rep. 4, 4026. 10.1038/srep04026 24504028 PMC4168524

[B10] Del Campo J. M. Matulonis U. A. Malander S. Provencher D. Mahner S. Follana P. (2019). Niraparib Maintenance Therapy in Patients with Recurrent Ovarian Cancer after a Partial Response to the Last Platinum-Based Chemotherapy in the ENGOT-OV16/NOVA Trial. Jco 37 (32), 2968–2973. 10.1200/jco.18.02238 PMC6839909 31173551

[B11] Fragoso-Ontiveros V. Velázquez-Aragón J. A. Nuñez-Martínez P. M. de la Luz Mejía-Aguayo M. Vidal-Millán S. Pedroza-Torres A. (2019). Mexican BRCA1 Founder Mutation: Shortening the Gap in Genetic Assessment for Hereditary Breast and Ovarian Cancer Patients. PLoS One 14 (9), e0222709. 10.1371/journal.pone.0222709 31545835 PMC6756553

[B12] Gallardo-Rincón D. Alamilla-García G. Montes-Servín E. Morales-Vázquez F. Cano-Blanco C. Coronel-Martínez J. (2019). Experiencia con el uso de olaparib en pacientes con cáncer de ovario. Gac. Med. Mex. 155 (6), 585–589. 10.24875/GMM.19005494 31787769

[B13] Gallardo-Rincón D. Álvarez-Gómez R. M. Montes-Servín E. Toledo-Leyva A. Montes-Servín E. Michel-Tello D. (2020). Clinical Evaluation of BRCA1/2 Mutation in Mexican Ovarian Cancer Patients. Transl. Oncol. 13 (2), 212–220. 10.1016/j.tranon.2019.11.003 31869745 PMC6931216

[B14] Ghose A. Moschetta M. Pappas-Gogos G. Sheriff M. Boussios S. (2021). Genetic Aberrations of DNA Repair Pathways in Prostate Cancer: Translation to the Clinic. Ijms 22 (18), 9783. 10.3390/ijms22189783 34575947 PMC8471942

[B15] González-Martín A. Pothuri B. Vergote I. DePont Christensen R. Graybill W. Mirza M. R. (2019). Niraparib in Patients with Newly Diagnosed Advanced Ovarian Cancer. N. Engl. J. Med. 381 (25), 2391–2402. 10.1056/nejmoa1910962 31562799

[B16] Hennessy B. T. J. Timms K. M. Carey M. S. Gutin A. Meyer L. A. Flake D. D. 2nd (2010). Somatic Mutations in BRCA1 and BRCA2 Could Expand the Number of Patients that Benefit from Poly (ADP Ribose) Polymerase Inhibitors in Ovarian Cancer. Jco 28 (22), 3570–3576. 10.1200/jco.2009.27.2997 PMC2917312 20606085

[B17] James P. A. Sawyer S. Boyle S. Young M.-A. Kovalenko S. Doherty R. (2015). Large Genomic Rearrangements in the Familial Breast and Ovarian Cancer Gene BRCA1 Are Associated with an Increased Frequency of High Risk Features. Fam. Cancer 14 (2), 287–295. 10.1007/s10689-015-9785-0 25678442

[B18] Judkins T. Hendrickson B. C. Deffenbaugh A. M. Eliason K. Leclair B. Norton M. J. (2005). Application of Embryonic Lethal or Other Obvious Phenotypes to Characterize the Clinical Significance of Genetic Variants Found in Trans with Known Deleterious Mutations. Cancer Res. 65 (21), 10096–10103. 10.1158/0008-5472.can-05-1241 16267036

[B19] Judkins T. Rosenthal E. Arnell C. Burbidge L. A. Geary W. Barrus T. (2012). Clinical Significance of Large Rearrangements in BRCA1 and BRCA2. Cancer 118 (21), 5210–5216. 10.1002/cncr.27556 22544547 PMC3532625

[B20] Kwong A. Chen J. Shin V. Y. Ho J. C. W. Law F. B. F. Au C. H. (2015). The Importance of Analysis of Long-Range Rearrangement of BRCA1 and BRCA2 in Genetic Diagnosis of Familial Breast Cancer. Cancer Genet. 208 (9), 448–454. 10.1016/j.cancergen.2015.05.031 26271414

[B21] Martínez-Treviño D. A. León-Cachón R. B. R. Villarreal-Garza C. Aguilar Y Méndez D. Aguilar-Martínez E. Barrera-Saldaña H. A. (2018). A novel method to detect the Mexican founder mutation BRCA1 ex9-12del associated with breast and ovarian cancer using quantitative polymerase chain reaction and TaqMan® probes. Mol. Med. Rep. 18 (2), 1531–1537. 10.3892/mmr.2018.9141 29901183 PMC6072190

[B22] Moore K. Colombo N. Scambia G. Kim B.-G. Oaknin A. Friedlander M. (2018). Maintenance Olaparib in Patients with Newly Diagnosed Advanced Ovarian Cancer. N. Engl. J. Med. 379 (26), 2495–2505. 10.1056/nejmoa1810858 30345884

[B23] Oliver J. Quezada Urban R. Franco Cortés C. A. Díaz Velásquez C. E. Montealegre Paez A. L. Pacheco-Orozco R. A. (2019). Latin American Study of Hereditary Breast and Ovarian Cancer LACAM: A Genomic Epidemiology Approach. Front. Oncol. 9, 1429. 10.3389/fonc.2019.01429 31921681 PMC6933010

[B24] Ossa C. A. Torres D. (2016). Founder and Recurrent Mutations in BRCA1 and BRCA2 Genes in Latin American Countries: State of the Art and Literature Review. Oncologist 21 (7), 832–839. 10.1634/theoncologist.2015-0416 27286788 PMC4943386

[B25] Pennington K. P. Walsh T. Harrell M. I. Lee M. K. Pennil C. C. Rendi M. H. (2014). Germline and Somatic Mutations in Homologous Recombination Genes Predict Platinum Response and Survival in Ovarian, Fallopian Tube, and Peritoneal Carcinomas. Clin. Cancer Res. 20 (3), 764–775. 10.1158/1078-0432.ccr-13-2287 24240112 PMC3944197

[B26] Pujade-Lauraine E. Ledermann J. A. Selle F. Gebski V. Penson R. T. Oza A. M. (2017). Olaparib Tablets as Maintenance Therapy in Patients with Platinum-Sensitive, Relapsed Ovarian Cancer and a BRCA1/2 Mutation (SOLO2/ENGOT-Ov21): a Double-Blind, Randomised, Placebo-Controlled, Phase 3 Trial. Lancet Oncol. 18 (9), 1274–1284. 10.1016/S1470-2045(17)30469-2 28754483

[B27] Quezada Urban R. Díaz Velásquez C. E. Gitler R. Rojo Castillo M. P. Sirota Toporek M. Figueroa Morales A. (2018). Comprehensive Analysis of Germline Variants in Mexican Patients with Hereditary Breast and Ovarian Cancer Susceptibility. Cancers (Basel) 10 (10), 361. 10.3390/cancers10100361 PMC6211045 30262796

[B28] Randall M. Burgess K. Buckingham L. Usha L. (2020). Exceptional Response to Olaparib in a Patient with Recurrent Ovarian Cancer and an Entire BRCA1 Germline Gene Deletion. J. Natl. Compr. Canc Netw. 18 (3), 223–228. 10.6004/jnccn.2019.7378 32135515

[B29] Sluiter M. D. van Rensburg E. J. (2011). Large Genomic Rearrangements of the BRCA1 and BRCA2 Genes: Review of the Literature and Report of a Novel BRCA1 Mutation. Breast Cancer Res. Treat. 125, 325–349. 10.1007/s10549-010-0817-z 20232141

[B30] The Global Cancer Observatory (2020). GLOBOCAN 2020. [Internet]. Lyon CEDEX 08, France. The International Agency for Research on Cancer (IARC) Is the Specialized Cancer Agency of the World Health Organization. The Objective of the IARC Is to Promote International Collaboration in Cancer Research 2020. Available at: https://gco.iarc.fr/ (Accessed July 30, 2021).

[B31] Toss A. Tomasello C. Razzaboni E. Contu G. Grandi G. Cagnacci A. (2015). Hereditary Ovarian Cancer: Not Only BRCA 1 and 2 Genes. Biomed. Res. Int. 2015, 341723. 10.1155/2015/341723 26075229 PMC4449870

[B32] Vaca-Paniagua F. Alvarez-Gomez R. M. Fragoso-Ontiveros V. Vidal-Millan S. Herrera L. A. Cantú D. (2012). Full-exon Pyrosequencing Screening of BRCA Germline Mutations in Mexican Women with Inherited Breast and Ovarian Cancer. PLoS One 7 (5), e37432. 10.1371/journal.pone.0037432 22655046 PMC3360054

[B33] Villarreal-Garza C. Alvarez-Gómez R. M. Pérez-Plasencia C. Herrera L. A. Herzog J. Castillo D. (2015). Significant Clinical Impact of recurrentBRCA1andBRCA2mutations in Mexico. Cancer 121 (3), 372–378. 10.1002/cncr.29058 25236687 PMC4304938

[B34] Villarreal-Garza C. Weitzel J. N. Llacuachaqui M. Sifuentes E. Magallanes-Hoyos M. C. Gallardo L. (2015). The Prevalence of BRCA1 and BRCA2 Mutations Among Young Mexican Women with Triple-Negative Breast Cancer. Breast Cancer Res. Treat. 150 (2), 389–394. 10.1007/s10549-015-3312-8 25716084 PMC4532439

[B35] Wang X. Hu N. Cui L. Si Y. Yue J. Zheng F. (2022). Durable Disease-free Survival in a Patient with Metastatic Triple-Negative Breast Cancer Treated with Olaparib Monotherapy. Curr. Cancer Drug Targets [Epub ahead of print]. 10.2174/1568009622666220214092207 PMC9906627 35156571

[B36] Weaver A. N. Yang E. S. (2013). Beyond DNA Repair: Additional Functions of PARP-1 in Cancer. Front. Oncol. 3, 290. 10.3389/fonc.2013.00290 24350055 PMC3841914

[B37] Weitzel J. N. Lagos V. Blazer K. R. Nelson R. Ricker C. Herzog J. (2005). Prevalence of BRCA Mutations and Founder Effect in High-Risk Hispanic Families. Cancer Epidemiol. Biomarkers Prev. 14 (7), 1666–1671. 10.1158/1055-9965.epi-05-0072 16030099

[B38] Weitzel J. N. Lagos V. I. Herzog J. S. Judkins T. Hendrickson B. Ho J. S. (2007). Evidence for Common Ancestral Origin of a Recurring BRCA1 Genomic Rearrangement Identified in High-Risk Hispanic Families. Cancer Epidemiol. Biomarkers Prev. 16 (8), 1615–1620. 10.1158/1055-9965.epi-07-0198 17646271

[B39] Weitzel J. N. Clague J. Martir-Negron A. Ogaz R. Herzog J. Ricker C. (2013). Prevalence and Type ofBRCAMutations in Hispanics Undergoing Genetic Cancer Risk Assessment in the Southwestern United States: A Report from the Clinical Cancer Genetics Community Research Network. Jco 31 (2), 210–216. 10.1200/jco.2011.41.0027 PMC3532393 23233716

[B40] Zhang S. Royer R. Li S. McLaughlin J. R. Rosen B. Risch H. A. (2011). Frequencies of BRCA1 and BRCA2 Mutations Among 1,342 Unselected Patients with Invasive Ovarian Cancer. Gynecol. Oncol. 121 (2), 353–357. 10.1016/j.ygyno.2011.01.020 21324516

