# Supplementary material for: Clinical Benefits of Olaparib in Mexican Ovarian Cancer Patients With Founder Mutation BRCA1-Del ex9-12
Source: Front Genet. 2022 Jun 6;13:863956. doi: 10.3389/fgene.2022.863956 (PMC9207274; doi:10.3389/fgene.2022.863956)
Supplement: Supplementary file 1 [file DataSheet1.docx]

**Supplementary Material**

| **S1. Patients therapy characteristics** | | | | |
| --- | --- | --- | --- | --- |
| **Variable** | **Total**  **% (*N*=35)** | ***BRCA1***  **(Founder Mutation)** | **Other *BRCA* mutations** | ***P**** |
|  |  | **% (n=9)** | **% (n=26)** |  |
| **Previous CT** | |  |  |  |
| CBP + TXL | 60 (21/35) | 55.6 (5/9) | 61.5 (16/26) | 0.287 |
| CDDP + TXL | 14.3 (5/35) | 11.1 (1/9) | 15.4 (4/26) |  |
| CBP + GMZ | 2.9 (1/35) | 0 (0(9) | 3.8 (1/26) |  |
| CDDP + GMZ | 8.6 (3/35) | 0 (0(9) | 11.5 (3/26) |  |
| CBP | 2.9 (1/35) | 11.1 (1/9) | 0 (0/26) |  |
| CBP + TXL + BVZ | 11.5 (4/35) | 22.22 (2/9) | 7.6 (2/26) |  |
| **Response to previous CT** | |  |  |  |
| Complete | 25.7 (9/35) | 33.33 (3/9) | 23.1 (6/26) | 0.758 |
| Partial | 65.7 (23/35) | 55.6 (5/9) | 69.2 (18/26) |  |
| Stable disease | 8.6 (3/35) | 11.11 (1/9) | 7.7 (2/26) |  |
| **Line of olaparib maintenance** | |  |  |  |
| 1 | 28.6 (10/35) | 11.11 (1/9) | 34.6 (9/26) | 0.399 |
| 2 | 20 (7/35) | 33.33 (3/9) | 15.4 (4/26) |  |
| 3 | 14.3 (5/35) | 22.22 (2/9) | 11.5 (3/26) |  |
| ≥4 | 37.1 (14/35) | 33.33 (3/9) | 38.5 (11/26) |  |
| **Platinum-based therapy** | |  |  |  |
| NA | 28.6 (10/35) | 11.11 (1/9) | 34.6 (9/26) | 0.361 |
| 6-12 months | 40 (14/35) | 55.6 (5/9) | 34.6 (9/26) |  |
| + 12 months | 31.4 (11/35) | 33.33 (3/9) | 30.8 (8/26) |  |
| **Dose adjustment for toxicity** | | |  |  |
| Negative | 51.4 (18/35) | 66.7 (6/9) | 46.2 (12/26) | 0.289 |
| Positive | 48.6 (17/35) | 33.33 (3/9) | 53.8 (14/26) |  |
| Hematological | 76.5 (13/17) | 66.7 (2/3) | 78.6 (11/14) | 0.659 |
| Gastrointestinal | 23.5 (4/17) | 33.3 (1/3) | 21.4 (3/14) |  |
| **BRCA location** | | |  |  |
| OCCR | 68.6 (24/35) | 100 (9/9) | 57.7 (15/26) | **0.018** |
| BCCR | 31.4 (11/35) | 0 (0(9) | 42.3 (11/26) |  |
| Abbreviations: CT: CT: Chemotherapy; CBP: Carboplatin; TXL: Taxol; CDDP: Cisplatin; GMZ: Gemcitabine; BVZ: Bevacizumab; OCCR: Ovarian Cancer Cluster Regions; BCCR: Breast Cancer Cluster Regions. *Chi-square (X2) test. | | | | |

| **S2. PFS according to baseline characteristics** | | | | | | | |
| --- | --- | --- | --- | --- | --- | --- | --- |
| **Variable** | **Total** | | | ***BRCA1*  (Founder Mutation)** | | **Other *BRCA* mutations** | |
|  | **N** | **Median (95% CI)** | ***P**** | **n** | **Median (95% CI)** | **n** | **Median (95% CI)** |
| **Total** | 35 | 12.87 (8.82 - 16.92) PFS at 36mos 36% |  | 9 | NR (NR - NR) PFS at 36mos 73% | 26 | 11.59 (10.43 - 12.75 PFS at 36mo 23% |
| **Age at diagnosis (years)** | | |  |  |  |  |  |
| ≤50 | 15 | NR (NR - NR) PFS at 36mos 58% | 0.434 | 4 | NR (NR - NR) PFS at 36mos 75% | 11 | NR (NR - NR) PFS at 36mo 50% |
| ≥51 | 20 | 12.18 (10.47 - 13.90) PFS at 36mos 22% |  | 5 | NR (NR - NR) PFS at 36mos 67% | 15 | 11.59 (8.31 - 14.88) PFS at 36mos 8% |
| **Histology** |  |  |  |  |  |  |  |
| HGS | 33 | 14.68 (10.64 - 18.72) PFS at 36mos 38% | -- | 9 | NR (NR - NR) PFS at 36mos 73% | 24 | 12.02 (10.67 - 13.37) PFS at 36mos 25% |
| Adenocarcinoma | 1 | -- |  | 0 | -- | 1 | -- |
| Others | 1 | -- |  | 0 | -- | 1 | -- |
| **Stage** |  |  |  |  |  |  |  |
| I - II | 4 | 11.30 (0.00 - 23.89) PFS at 36mos 38% | 0.113 | 0 | -- | 4 | 11.30 (0.00 - 23.89) PFS at 36mos 38% |
| IIIA - B | 3 | 17.97 (7.50 - 22.02) PFS at 36mos 0% |  | 2 | NR (NR - NR) PFS at 36mos 0% | 1 | -- |
| IIIC | 19 | 11.59 (8.28 - 14.91) PFS at 36mos 25% |  | 3 | NR (NR - NR) PFS at 36mos 67% | 16 | 11.26 (7.53 - 15.00) PFS at 36mos 16% |
| IV | 9 | NR (NR - NR) PFS at 36mos 71% |  | 4 | NR (NR - NR) PFS at 36mos 100% | 5 | 15.31 (9.95 - 21.83) PFS at 36mos 0% |
| **DM** |  |  |  |  |  |  |  |
| Negative | 31 | 14.68 (8.72 – 20.64) PFS at 36mos 38% | 0.798 | 9 | NR (NR - NR) PFS at 36mos 73% | 22 | 11.30 (8.67 - 13.93) PFS at 36mos 25% |
| Positive | 4 | 12.18 (4.65 - 19.72) PFS at 36mos 0% |  | 0 | -- | 4 | 12.18 (4.65 - 19.72) PFS at 36mos 0% |
| **SAH** |  |  |  |  |  |  |  |
| Negative | 29 | 14.68 (8.85 - 20.51) PFS at 36mos 42% | 0.999 | 8 | NR (NR - NR) PFS at 36mos 86% | 21 | 11.30 (8.09 - 14.51) PFS at 36mos 24% |
| Positive | 6 | 12.02 (11.31 - 12.73) PFS at 36mos 0% |  | 1 | -- | 5 | 12.02 (11.11 - 12.94) PFS at 36mos 0% |
| **CFH** |  |  |  |  |  |  |  |
| Negative | 4 | 11.59 (6.76 - 16.42) PFS at 36mos 0% | 0.595 | 0 | -- | 4 | 11.59 (6.76 - 16.42) PFS at 36mos 0% |
| Positive | 31 | 14.68 (6.48 - 22.88) PFS at 36mos 43% |  | 9 | NR (NR - NR) PFS at 36mos 100% | 22 | 11.30 (7.86 - 14.73) PFS at 36mos 30% |
| **Ovarian CFH** |  |  |  |  |  |  |  |
| Negative | 23 | 12.18 (10.39 - 13.98) PFS at 36mos 31% | 0.365 | 7 | NR (NR - NR) PFS at 36mos 69% | 16 | 11.30 (7.25 - 15.34) PFS at 36mos 44% |
| Positive | 12 | NR (NR – NR) PFS at 36mos 50% |  | 2 | NR (NR - NR) PFS at 36mos 100% | 10 | 14.68 (6.66 - 22.71) PFS at 36mos 14% |
| **Breast CFH** |  |  |  |  |  |  |  |
| Negative | 12 | 11.59 (8.73 - 14.45) PFS at 36mos 0% | **0.036** | 1 | -- | 11 | 11.59 (8.72 - 14.47) PFS at 36mos 0% |
| Positive | 23 | 17.97 (0.00 - 42.93) PFS at 36mos 49% |  | 8 | NR (NR - NR) PFS at 36mos 74% | 15 | 12.87 (5.48 - 20.27) PFS at 36mos 35% |
| **Other CFH** |  |  |  |  |  |  |  |
| Negative | 20 | 12.02 (9.88 - 14.16) PFS at 36mos 30% | 0.721 | 4 | NR (NR - NR) PFS at 36mos 100% | 16 | 11.30 (6.92 - 15.67) PFS at 36mos 0% |
| Positive | 15 | 17.97 (0.00 - 47.96) PFS at 36mos 45% |  | 5 | NR (NR - NR) PFS at 36mos 50% | 10 | 12.18 (6.21 - 18.16) PFS at 36mos 42% |
| Pancreatic | 4 | 6.66 (4.35 - 17.98) PFS at 36mos 0% | 0.442 | 2 | NR (NR - NR) PFS at 36mos 50% | 2 | -- |
| Prostate | 5 | 9.69 (2.21 - 17.16) PFS at 36mos 0% |  | 1 | -- | 4 | 6.20 (0.00 - 12.87) PFS at 36mos 0% |
| Gastrointestinal | 6 | 39.62 (0.00 - 86.10) PFS at 36mos 61% |  | 5 | NR (NR - NR) PFS at 36mos  50% | 4 | 39.62 (0.00 - 83.52) PFS at 36mos 67% |
| **Double primary malignancy** | | | |  |  |  |  |
| Negative | 27 | 12.18 (10.07 - 14.30) PFS at 36mos 40% | 0.76 | 5 | NR (NR - NR) PFS at 36mos 100% | 22 | 11.30 (8.62 - 13.98) PFS at 36mos 25% |
| Positive | 8 | 14.68 (3.95 - 25.41) PFS at 36mos 23% |  | 4 | NR (NR - NR) PFS at 36mos 33% | 4 | 14.68 (4.45 - 18.81) PFS at 36mos 0% |
| Abbreviations: HGS: High-grade serous; DM: Diabetes Mellitus; SAH: Systemic Arterial Hypertension CFH: Cancer Family History. *Kaplan–Meier method with log-rank or Breslow tests. | | | | | | | |

| **S3. PFS according to patients’ therapy characteristics** | | | | | | | |
| --- | --- | --- | --- | --- | --- | --- | --- |
| **Variable** | **Total** | | | ***BRCA1*  (Founder Mutation)** | | **Other *BRCA* mutations** | |
|  | **N** | **Median (95% CI)** | ***P**** | **n** | **Median (95% CI)** | **n** | **Median (95% CI)** |
| **Total** | 35 | 12.87 (8.82 - 16.92) PFS at 36mos 36% |  | 9 | NR (NR - NR) PFS at 36mos 73% | 26 | 11.59 (10.43 - 12.75 PFS at 36mo 23% |
| **Response to previous CT** | | |  |  |  |  |  |
| Complete | 9 | 17.97 (10.19 - 25.74) PFS at 36mos 44% | 0.452 | 3 | NR (NR - NR) PFS at 36mos 67% | 6 | 11.30 (4.55 - 18.04) PFS at 36mos 34% |
| Partial | 23 | 12.18 (10.46 - 13.91) PFS at 36mos 35% |  | 5 | NR (NR - NR) PFS at 36mos 75% | 18 | 11.02 (10.88 - 13.16) PFS at 36mos 23% |
| Stable disease | 3 | 6.20 (2.05 - 10.36) PFS at 36mos 0% |  | 1 | -- | 2 | 3.61 (2.36 - 7.45) PFS at 36mos 0% |
| **Line of Olaparib maintenance** | | |  |  |  |  |  |
| 1 | 10 | 12.87 (9.67 - 16.08) PFS at 36mos 0% | 0.390 | 1 | -- | 9 | 12.87 (9.67 - 16.08) PFS at 36mos 0% |
| 2 | 7 | 39.62 (0.00 - 87.83) PFS at 36mos 72% |  | 3 | NR (NR - NR) PFS at 36mos 67% | 4 | 39.62 (0.00 - 89.64) PFS at 36mos 75% |
| 3 | 5 | 15.31 (6.70 - 23.91) PFS at 36mos 21% |  | 2 | NR (NR - NR) PFS at 36mos 50% | 3 | 11.30 (0.00 - 24.76) PFS at 36mos 0% |
| ≥4 | 13 | 11.59 (7.27 - 15.91) PFS at 36mos 23% |  | 3 | NR (NR - NR) PFS at 36mos 100% | 10 | 8.34 (3.25 - 13.43) PFS at 36mos 0% |
| **By clinical trials criteria** | | |  |  |  |  |  |
| SOLO1 (First line maintenance) | 10 | 12.87 (9.67 - 16.08) PFS at 36mos 0% | 0.713 | 1 | -- | 9 | 12.87 (9.67 - 16.08) PFS at 36mos 0% |
| SOLO2 (Recurrent disease) | 25 | 12.18 (7.14 - 17.22) PFS at 36mos 36% |  | 8 | NR (NR - NR) PFS at 36mos 73%% | 17 | 11.30 (6.92 - 15.67) PFS at 36mos 18% |
| **Platinum-based therapy** | | |  |  |  |  |  |
| NA | 10 | 12.87 (9.67 - 16.08) PFS at 36mos 0% | 0.912 | 1 | -- | 9 | 12.87 (9.67 - 16.08) PFS at 36mos 0% |
| 6-12 months | 14 | 11.59 (7.32 - 15.87) PFS at 36mos 36% |  | 5 | NR (NR - NR) PFS at 36mos 80% | 9 | 9.69 (5.75 - 13.62) PFS at 36mos 0% |
| + 12 months | 11 | 15.31 (8.11 - 22.50) PFS at 36mos 35% |  | 3 | NR (NR - NR) PFS at 36mos 67% | 8 | 11.30 (0.00 - 22.77) PFS at 36mos 25% |
| **Dose adjustment for toxicity** | | | |  |  |  |  |
| Negative | 18 | 14.68 (3.85 - 25.52) PFS at 36mos 40% | 0.698 | 6 | NR (NR - NR) PFS at 36mos 60% | 12 | 11.30 (6.78 - 15.82) PFS at 36mos 29% |
| Positive | 17 | 12.18 (10.46 - 13.91) PFS at 36mos 0% |  | 3 | NR (NR - NR) PFS at 36mos 100% | 14 | 11.59 (7.32 - 15.87) PFS at 36mos 0% |
| Hematological | 13 | 12.18 (10.68 – 13.69) PFS at 36mos 0% | 0.653 | 2 | NR (NR - NR) PFS at 36mos 100% | 11 | 12.02 (9.33 - 14.71) PFS at 36mos 0% |
| Gastrointestinal | 4 | 7.09 (0.00 - 20.01) PFS at 36mos 0% |  | 1 | -- | 3 | 7.09 (0.00 - 15.03) PFS at 36mos 0% |
| **BRCA location** | | |  |  |  |  |  |
| OCCR | 24 | 15.31 (7.82 - 22.79) PFS at 36mos 42% | 0.627 | 9 | NR (NR - NR) PFS at 36mos 73% | 15 | 11.26 (5.04 - 17.48) PFS at 36mos 21% |
| BCCR | 11 | 11.59 (9.08 - 14.11) PFS at 36mos 0% |  | 0 | -- | 11 | 11.59 (9.08 - 14.11) PFS at 36mos 0% |
| Abbreviations: Ovarian Cancer Cluster Regions (OCCR); Breast Cancer Cluster Regions (BCCR). *Kaplan–Meier method with log-rank or Breslow tests. | | | | | | | |

| **S4. Toxicity adverse events in patients that received Olaparib maintenance therapy (CTCAE.v4)** | | | | |
| --- | --- | --- | --- | --- |
| **Toxicity/ Adverse Event** | **Total OC patients** | | | |
|  | **N=35** | | | |
|  | G1 | G2 | G3 | G4 |
| **Hematological toxicity** | | | | |
| Anemia | 0 | 5 | 7 | 3 |
| Neutropenia | 0 | 0 | 1 | 0 |
| **Gastrointestinal toxicity** | | | | |
| Nausea | 0 | 2 | 1 | 0 |
| Dysgeusia | 0 | 1 | 0 | 0 |
| **Others** | | | | |
| Fatigue | 14 | 0 | 0 | 0 |
| Pneumonitis | 0 | 1 | 0 | 0 |
| Abbreviations: CTCAE= National Cancer Institute Common Terminology Criteria for Adverse Events | | | | |
